# Metabolic dysfunction in female mice with disruption of 5α-reductase 1

**DOI:** 10.1530/JOE-16-0125

**Published:** 2016-11-11

**Authors:** Dawn E W Livingstone, Emma M Di Rollo, Tracy C-S Mak, Karen Sooy, Brian R Walker, Ruth Andrew

**Affiliations:** 1 University/British Heart Foundation Centre for Cardiovascular ScienceUniversity of Edinburgh, Queen’s Medical Research Institute, Edinburgh, UK; 2 Centre for Integrative PhysiologyUniversity of Edinburgh, Edinburgh, UK

**Keywords:** glucocorticoid, steroid metabolising hormones, metabolism, obesity

## Abstract

5α-Reductases irreversibly catalyse A-ring reduction of pregnene steroids, including glucocorticoids and androgens. Genetic disruption of 5α-reductase 1 in male mice impairs glucocorticoid clearance and predisposes to glucose intolerance and hepatic steatosis upon metabolic challenge. However, it is unclear whether this is driven by changes in androgen and/or glucocorticoid action. Female mice with transgenic disruption of 5α-reductase 1 (5αR1-KO) were studied, representing a ‘low androgen’ state. Glucocorticoid clearance and stress responses were studied in mice aged 6 months. Metabolism was assessed in mice on normal chow (aged 6 and 12 m) and also in a separate cohort following 1-month high-fat diet (aged 3 m). Female 5αR1-KO mice had adrenal suppression (44% lower AUC corticosterone after stress), and upon corticosterone infusion, accumulated hepatic glucocorticoids (~27% increased corticosterone). Female 5αR1-KO mice aged 6 m fed normal chow demonstrated insulin resistance (~35% increased area under curve (AUC) for insulin upon glucose tolerance testing) and hepatic steatosis (~33% increased hepatic triglycerides) compared with controls. This progressed to obesity (~12% increased body weight) and sustained insulin resistance (~38% increased AUC insulin) by age 12 m. Hepatic transcript profiles supported impaired lipid β-oxidation and increased triglyceride storage. Female 5αR1-KO mice were also predisposed to develop high-fat diet-induced insulin resistance. Exaggerated predisposition to metabolic disorders in female mice, compared with that seen in male mice, after disruption of 5αR1 suggests phenotypic changes may be underpinned by altered metabolism of glucocorticoids rather than androgens.

## Introduction

5α-Reductases catalyse the irreversible A-ring reduction of pregnene steroids and hence regulate the levels of active steroids and their metabolites within tissues (Russell & Wilson 1994). There are three isozymes of 5αRs, with types 1 and 2 being best characterised (Godoy *et al.* 2011). The 5α-dihydro metabolite (DHT) is a more potent androgen than testosterone. The 3α,5α-metabolites of glucocorticoids retain some anti-inflammatory properties of the original steroid (corticosterone in rodents and cortisol in humans), but lack many metabolic properties (McInnes *et al.* 2004, Yang *et al.* 2011). Given the potent effects of both glucocorticoids and androgens on metabolic homeostasis, there has been recent interest in the consequences of inhibition of 5αRs on metabolic health. This has implications for men receiving 5αR inhibitors for chronic treatment of prostatic disease or women receiving these drugs for hirsutism. Furthermore, 5α-reduction of steroid hormones is increased in obesity (Andrew *et al.* 1998, Fraser *et al.* 1999) and polycystic ovarian syndrome (PCOS) (Stewart *et al.* 1990).

5αR1 is the dominant isozyme in rodent metabolic tissues, and male mice with global genetic disruption of this enzyme (but not of 5αR2) develop obesity and insulin resistance, with increased susceptibility to liver steatosis, fibrosis and hepatocellular carcinoma (Dowman *et al.* 2013, Livingstone *et al.* 2015). These findings translate into humans, where dutasteride (a dual 5αR inhibitor) induces peripheral insulin resistance and hepatic lipid accumulation (Upreti *et al.* 2014, Hazlehurst *et al.* 2016). Questions remain as to whether these adverse effects result from changes in glucocorticoid and/or androgen signalling or indeed both. Insulin resistance is a feature of both excess glucocorticoid production, for example in Cushing’s Syndrome, and also low circulating testosterone with ageing (Cheung *et al.* 2015).

*In vivo* dissection of the distinct contributions of androgens and glucocorticoids to the metabolic phenotype in the 5αR1-KO mice is difficult. There is considerable overlap in the responses to glucocorticoid and androgens amongst transcripts of hepatic genes regulating carbohydrate and lipid homeostasis (Dowman *et al.* 2013). In Zucker rats, castration limits insulin resistance induced by 5αR inhibition by finasteride (a non-selective 5α-reductase inhibitor in rodents (Thigpen & Russell 1992)) but does not attenuate the induction of hepatic steatosis, implying that changes in glucocorticoid signalling may underpin non-alcoholic fatty liver disease in 5αR1 deficiency (Livingstone *et al.* 2015). Here, we investigate whether 5αR1 deficiency in female mice, representative of ‘a low androgen’ state, causes similar metabolic disturbances to males.

## Materials and methods

Chemicals were from Sigma unless stated, steroids from Steraloids (Newport, RI, USA) and HPLC-grade solvents from Thermo Fisher Scientific.

### Animal husbandry

Embryos (C57Bl6/SvEv/129) with targeted disruption of 5αR1 (Jackson Laboratory) (Mahendroo *et al.* 1996) were re-derived (Livingstone *et al.* 2014) and allowed free access to double-filtered drinking water and standard chow (7.4% fat, 4% sucrose; RM1, Special Diet Services, Witham, Essex, UK), unless otherwise stated. At the end of experiments animals were decapitated (08:00–11:00 h), trunk blood was collected and tissues were wet weighed and either snap-frozen or fixed in formalin (adrenal, thymus). All experiments were conducted under authority of UK Home Office licence and were subject to internal ethical review by named veterinary surgeons at the University of Edinburgh.

### HPA function in 5αR1-deficient mice

Basal nadir (08:00 h) and zenith (19:00 h) corticosterone was assessed in blood (mice aged 12 m, *n* = 12/group), collected by tail nick. A separate cohort of mice (*n* = 12/group) underwent acute restraint stress (15 min), and serial blood samples were taken up to 90 min after the onset of stress (Livingstone *et al.* 2014). Mice were culled one week after testing.

### Glucocorticoid clearance in 5αR1-deficient mice

Female 5αR1-KO mice and wild-type (WT) controls (age 3–4 m, *n* = 7–8/group) underwent bilateral adrenalectomy to remove endogenous glucocorticoids and had a fixed rate corticosterone infusion by Alzet osmotic mini-pump (100 µg corticosterone/day in 1:1 DMSO:propylene glycol; Charles River) to assess steady state corticosterone clearance rate (Livingstone *et al.* 2014).

### Metabolic function in 5αR1-deficient mice

Female WT and 5αR1-KO mice were fed standard chow from weaning, and at age 6 months (*n* = 19–20/group), free ambulatory behaviour was assessed in home cages containing single mice, in a recording frame (Linton Instruments, Diss, Norfolk, UK) overnight (14.5 h) and quantified using AMON software. Animals were re-housed in groups, after testing. One week later, mice underwent glucose tolerance testing (GTT: 2 g glucose/kg body weight i.p., after a 6-h fast) and were culled two days later. Further mice (littermates of male mice previously reported (Livingstone *et al.* 2015)) were aged to 12 m, subjected to GTT as above and culled (*n* = 15/group).

A further cohort of mice aged 3 m were fed high-fat sucrose (58% kcal fat and 13% kcal sucrose) or control diet (10.5% kcal fat, 0% kcal sucrose; Research Diets Inc, New Brunswick, NJ, USA) for 4 weeks, subjected to GTT as above and culled (*n* = 10–18/group).

### Laboratory analyses

In clearance studies, corticosterone in plasma, liver and brain was quantified by liquid chromatography tandem mass spectrometry (Livingstone *et al.* 2014). Otherwise, corticosterone was measured by ELISA (Enzo Life Sciences, Exeter, Devon, UK), testosterone by radioimmunoassay (Corker & Davidson 1978), insulin by ELISA (Crystal Chem Inc, Downers Grove, IL, USA), triglycerides and glucose (Thermo Fisher Scientific) and NEFAs (Zen-Bio, Research Triangle Park, NC, USA) spectrophotometrically (Livingstone *et al.* 2015). Hepatic glucocorticoid-metabolising enzyme activity was measured as described previously (Livingstone *et al.* 2014). Transcript abundance was measured by quantitative PCR using the standard curve method, except for *Crh* (corticotrophin-releasing hormone), which was measured by *in situ* hybridisation (Livingstone *et al.* 2014). PCR assay details and gene abbreviations are given in Supplementary Table 1 (see section on supplementary data given at the end of this article). qPCR results were normalised for the abundance of reference genes (liver; mean of 18S ribosomal RNA and *Actb*: hypothalamus; *Gapdh* and pituitary; *Tbp*), which were not different between groups.

### Statistical analysis

Data are represented as mean ± s.e.m. Groups were compared by Student’s *t* tests or ANOVA (with Fisher’s *post hoc* where appropriate), as indicated in the text.

## Results

### Glucocorticoid clearance in female 5αR1-KO mice

Deficiency of 5αR1 in the liver was confirmed by qPCR (Table 1), with mRNA for 5αR1 below the limit of quantitation in 5αR1-KO mice meaning transcript abundance was at least 100-fold less than that in wild-type mice. Other enzymes involved with hepatic clearance of glucocorticoids (11β-hydroxysteroid-dehydrogenase 1,5β-reductase) were not altered by genotype (Table 1).

**Table 1:** Morphometry in female 5αR1-KO and wild-type mice, aged 6 and 12 months.

	**6 months**		**12 months**	
	**WT**	**5αR1-KO**	**WT**	**5αR1-KO**
Endocrine parameters				
Adrenal gland (mg)			2.3 ± 0.3	2.3 ± 0.3
Thymus (mg)			34.1 ± 2.3	34.9 ± 2.9
Plasma testosterone (pg/mL)			66 ± 10	123 ± 24*
Liver glucocorticoid metabolism				
11βHSD1 velocity (nmol/mg/h)			20.1 ± 2.3	16.5 ± 1.1
5β-Reductase velocity (pmol/mg/h)			238 ± 17	254 ± 12
*Hsd11b1* (11βHSD1) transcript^#^			0.81 ± 0.08	0.85 ± 0.10
*Akr1d1* (5β-reductase) transcript^#^			0.77 ± 0.14	0.98 ± 0.09
*Srd5a1* (5α-reductase 1) transcript^#^			2.13 ± 0.21	<0.024*** (LOQ)
Metabolic parameters				
Body weight (g)	26.7 ± 0.53	28.4 ± 0.58*	32.4 ± 0.85	37.6 ± 1.91*
Food intake (g/day)	3.45 ± 0.19	3.10 ± 0.26	4.25 ± 0.10	4.12 ± 0.09
Liver (mg)	995 ± 64	1083 ± 59	1340 ± 49	1593 ± 49*
Omental adipose (mg)	30 ± 3	41 ± 19	55 ± 10	51 ± 10
Mesenteric adipose (mg)	391 ± 25	448 ± 26	499 ± 45	610 ± 90
Gonadal adipose (mg)	807 ± 65	1016 ± 57*	1066 ± 104	1953 ± 387*
Subcutaneous adipose (mg)	562 ± 53	718 ± 67	455 ± 39	684 ± 82*
Total adipose (g)	1.80 ± 0.12	2.21 ± .012*	2.08 ± 0.18	3.65 ± 0.64*
Hepatic triglycerides (µmol/g)	15.2 ± 1.5	20.2 ± 2.2*	23.9 ± 1.7	30.7 ± 2.6*

Data are mean ± s.e.m., compared by Student’s *t-*test. *P  < 0.05, ***P < 0.001 for effect of genotype. *N* = 10–15/group. LOQ = limit of quantitation. TAG = triglycerides. ^#^ denotes relative abundance of mRNA normalised to the mean of reference genes (18S ribosomal RNA and *Actb*), which did not differ between groups.

In adrenalectomised mice undergoing fixed infusion with corticosterone, circulating corticosterone levels were not significantly higher in 5αR1-KO than WT mice (166 ± 20 vs 157 ± 47 nM); however, corticosterone levels were elevated in 5αR1-KO mice in the liver (0.29 ± 0.028 vs 0.21 ± 0.013 nmol/g; *P* = 0.02) and in the brain (0.36 ± 0.052 vs 0.21 ± 0.023 pmol/g; *P* = 0.02).

### HPA axis response in female 5αR1-KO mice

**Figure 1:**
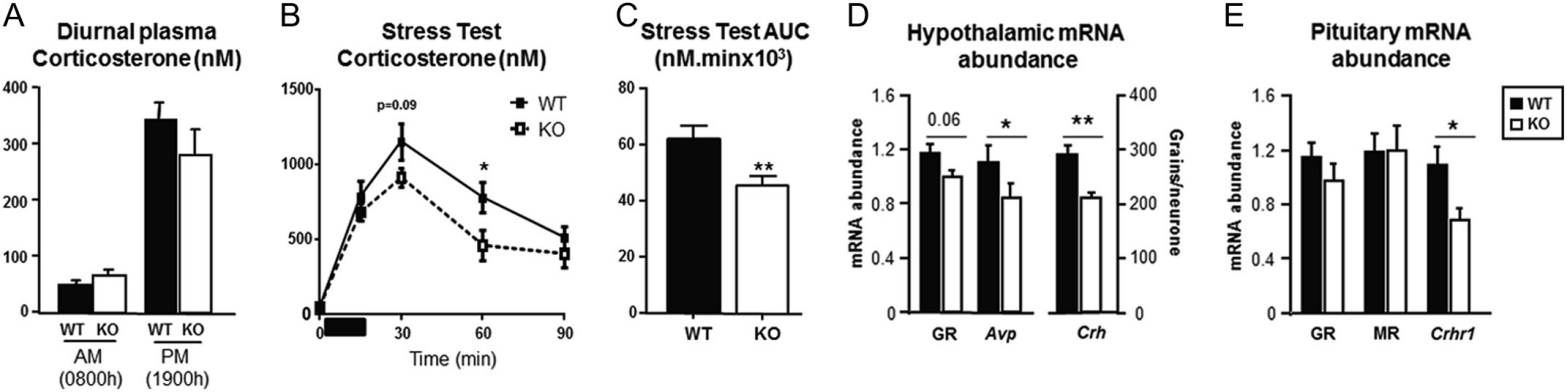
Dysregulation of the HPA axis in female mice deficient in 5α-reductase 1. (A) Female mice deficient in 5α-reductase 1 (KO) had normal plasma corticosterone at 08:00 h (nadir) and 19:00 h (zenith). (B) The increase in corticosterone in response to acute stress (duration of stress 15 min, indicated by dark bar) was attenuated in female mice deficient in 5αR1 (KO) compared with wild-type controls (WT), with a smaller area under the curve (C). (D) Abundance of mRNA transcript for *Avp* (arginine vasopressin) and *Crh* (corticotrophin-releasing hormone) were reduced in hypothalamus from KO mice, and there was a trend (*P* = 0.06) for reduced expression of glucocorticoid receptor (GR: *Nr3c1*). (E) Transcript for *Crhr1* (CRH-receptor 1) was lower in pituitaries from KO mice, but there was no difference in expression of GR or mineralocorticoid receptor (MR: *Nr3c2*). Data are mean ± s.e.m., *N* = 8–15 and compared by Student’s *t*-test (A, C, D and E), or ANOVA with *post hoc* comparisons (B). *P < 0.05 and **P < 0.01 vs WT. WT = filled bars/symbols and solid lines, KO = open bars/symbols and dashed lines.

Basal circulating corticosterone concentrations were not different between genotypes at either nadir (08:00 h) or zenith (19:00 h, Fig. 1A). After acute restraint, the AUC for the stress response was reduced in 5αR1-KO compared with WT mice (Fig. 1B, C). In the hypothalami from KO mice (Fig. 1D), there was reduced abundance of transcript for *Avp* and *Crh*, and a trend for a decrease in glucocorticoid receptor (GR: *Nr3c1*). *Crhr1* transcript abundance in the pituitary was reduced in KO mice (Fig. 1E), but there was no difference in either GR or mineralocorticoid receptor (MR: *Nr3c2*).

### Metabolic homeostasis in female 5αR1-KO mice

At 6 months of age, female 5αR1-KO mice were heavier than WT controls (Table 1) and had increased adipose tissue. Hepatic triglyceride content was higher in 5αR1-KO mice, but liver weight was not significantly increased. Total nocturnal ambulatory activity did not differ between genotypes (WT vs 5αR1-KO; total active time 7583 ± 531 vs 7362 ± 580 s; distance travelled 360 ± 41 vs 321 ± 60 m), and neither did food intake (Table 1).

**Figure 2:**
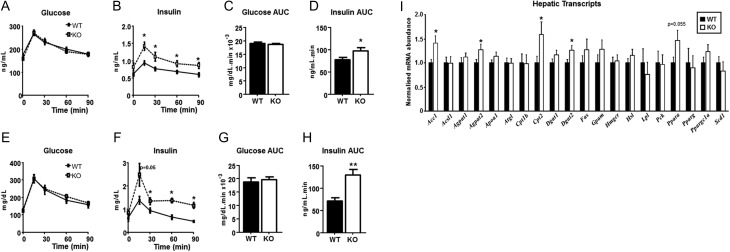
Metabolic dysfunction in female mice deficient in 5α-reductase 1. Female mice deficient in 5α-reductase 1 (KO) aged 6 m had normal fasting glucose and maintained their circulating glucose concentrations during glucose tolerance test (A); however, their insulin levels were higher during the test than wild-type mice (WT) (B). The area under the curve (AUC) for glucose was not altered between genotypes (C) but was increased for insulin in 5αR1-KO (D). At age 12 m, KO mice still had normal fasting glucose and response during GTT (E), with AUC unaffected by genotype (G). However, the area under the curve (AUC) for insulin was increased during the GTT in KO mice (F, H). Hepatic transcript profiling by quantitative PCR (I). Data are presented with WT data normalised to 1 for clarity, but statistical comparisons were made on raw data. *N* = 15–20/group. Data are mean ± s.e.m., compared by ANOVA in A, B, E and F, with *post hoc* comparisons in B and F, and by Student’s *t*-test in C, D, G, H and I. *P < 0.05, **P < 0.01 vs WT. WT = filled bars/symbols and solid lines, KO = open bars/symbols and dashed lines.

Fasting glucose was not different between genotypes (Fig. 2A), but there was a trend (*P* = 0.06) for higher fasting insulin in 5α-R1-KO mice (Fig. 2B). During the GTT, glucose levels were normal in 5α-R1-KO mice (Fig. 2A, C), but at the expense of higher plasma insulin (Fig. 2B, D).

As animals aged to 1 year, female 5αR1-KO mice remained heavier, gaining excess adipose throughout, but without consuming extra calories (Table 1). Liver weights and hepatic triglyceride content were higher in 5αR1-KO than WT mice (Table 1). On GTT, glucose was not different between genotypes (Fig. 2E, G), but again, insulin was elevated (Fig. 2F, H). NEFA suppression during GTT was not different between WT and 5αR1-KO mice (Δ^15–0 min^: 0.44 ± 0.08 vs 0.28 ± 0.06 µM). Hepatic transcripts of *Acc1*, *Agpat2*, *Cpt 2* and *Dgat2* (Fig. 2H) were increased in abundance in 5αR1-KO mice vs WT, with a trend towards increased *Ppar* α.

**Figure 3:**
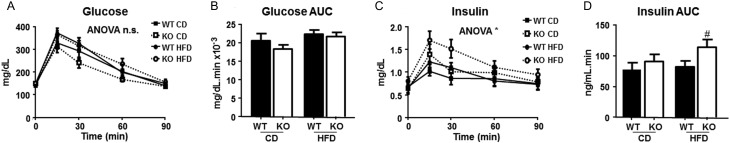
Insulin sensitivity in female mice deficient in 5α-reductase 1 aged 3 m after high-fat diet. Female mice deficient in 5α-reductase 1 (KO) and their wild-type controls (WT) aged 3m were fed high-fat (HFD) or control diet (CD). After 1 m of experimental diet, glucose concentrations during glucose tolerance test (GTT) (A) and the area under the curve for glucose during the GTT (B) were not affected by genotype or diet. However, the area under the curve (AUC) for insulin during the GTT was increased specifically in KO mice fed high-fat diet (C, D). *N* = 10–18/group, data are mean ± s.e.m., compared by two-way ANOVA with *post hoc* testing. *P < 0.05 on ANOVA, ^#^*P* < 0.05 compared with all other groups. n.s. is not significant. WT = filled bars/symbols and solid lines, KO = open bars/symbols and dashed lines.

Young (age 3 m) 5αR1-KO mice fed control diet were not heavier than WT (22.69 ± 0.63 vs 23.38 ± 0.61 g; *P* = 0.6), but when both genotypes were fed high-fat diet, 5αR1-KO were significantly heavier than WT (25.71 ± 0.61 vs 23.71 ± 0.57g; *P* <0.05). On GTT, glucose concentrations were not affected by genotype or diet (Fig. 3A, B). Insulin concentrations were not different in control diet-fed mice, but were significantly higher in the 5αR1-KO mice receiving high-fat diet vs WT (Fig. 3C, D).

## Discussion

Female 5αR1-KO mice are more susceptible to metabolic dysfunction than wild-type mice, both with ageing when fed normal chow and when fed high-fat high-sucrose diet. Female 5αR1-KO mice thus appear even more metabolically vulnerable than male 5αR1-KO mice; in two separate studies, male 5αR1-KO mice fed control diets for 6 months did not exhibit a metabolic phenotype (Dowman *et al.* 2013, Livingstone *et al.* 2015). Similar to male mice deficient in 5αR1 (Livingstone *et al.* 2014), female 5αR1-KO mice accumulated active glucocorticoid hormones within their livers and brains, indicating an impairment in clearance of corticosterone. Reduced glucocorticoid response to acute stress indicates that this alteration in clearance may be sufficient to incur adrenal suppression, as seen in male 5αR1-KO mice (Livingstone *et al.* 2014), and hypothalamic and pituitary transcript profiles are consistent with a decrease in HPA axis drive.

Disruption of 5αR1 in male mice adversely affects metabolism, but metabolic disturbance was only revealed after prolonged high-fat feeding (6 months), when they developed steatosis and insulin resistance, with exaggerated susceptibility to more severe liver disease (Dowman *et al.* 2013, Livingstone *et al.* 2015). However, female mice lacking 5αR1 developed similar features, with steatosis and glucose intolerance evident in chow-fed mice, without the requirement for stimulus of a metabolic dietary challenge. Female 5αR1-KO mice aged 6 months were overweight, with impaired insulin sensitivity and hepatic steatosis and this progressed further by 1 y. Similar to male mice, adipose insulin sensitivity was maintained, as evidenced by normal suppression of NEFAs during GTT, suggesting that liver may be the primary site underpinning metabolic disturbance in 5αR1-KO mice. In addition, just one month of high-fat diet in young female mice (3 m) was sufficient to reveal increased susceptibility to weight gain and insulin resistance in 5αR1-KO mice, whereas having no effect in WT mice.

The hepatic transcript profile of the female mice supported the concept of increased hepatic storage of lipid with reduced abundance of *Dgat2* mRNA. However, *Acc1*, generating malonyl CoA as a suppressor of β-oxidation, was also increased in females. Although this bore some similarities to the changes in male 5αR1-KO mice, direct gender comparisons cannot be made because previous males (Livingstone *et al.* 2015) were subject to different experimental conditions.

Female mice were studied to address the question of which hormonal change underpinned the phenotype of adverse metabolism in males, given both glucocorticoids and androgens (and indeed other hormones) are enzyme substrates with potent effects to regulate metabolism. Liver and brain glucocorticoid levels were increased in the female 5αR1-KO mice similar to males, by ~30–50% during chronic infusion of corticosterone (Livingstone *et al.* 2014). As with males, impaired hepatic clearance of corticosterone in female 5αR1-KO mice appeared of sufficient magnitude to reduce drive through the HPA axis, as supported by the reported reduced abundance of hypothalamic transcript encoding AVP and CRH and reduced pituitary expression of CRHR1 in 5αR1-KO mice and the subsequent adrenal suppression manifested in attenuated adrenal responses to stress compared with WT. Differences might have been anticipated to be less as female mice, unlike rats (Gustafsson & Stenberg 1974) and humans (Andrew *et al.* 1998, Fraser *et al.* 1999), have reduced expression of hepatic 5α-R1 mRNA compared with males. Furthermore, the presence of cyclical progesterone may have changed the competition between 5αR1 substrates for clearance (Andersson & Russell 1990, Normington & Russell 1992) between female vs male mice, with corticosterone binding with considerably less affinity than progesterone for 5αR1 (Normington & Russell 1992), but not 5β-reductase (Kondo *et al.* 1994).

The fact that female 5αR1-KO mice developed metabolic dysfunction at a younger age than their male counterparts, while sustaining similar changes in hepatic glucocorticoid levels, (Livingstone *et al.* 2014) points to the changes in androgen metabolism secondary to 5αR1 deficiency being protective. Indeed had androgens been the principle driver of adverse metabolism, the phenotype would be expected to be less pronounced in females than in males; however, the phenotype was more marked in females, pointing to the changes in androgen metabolism secondary to 5αR1 deficiency being protective. Female 5αR1-KO mice had increased testosterone vs WT (Table 1), but overall levels were more than ten-fold lower than those in males. The subtle increased testosterone was associated with increased oestradiol levels, likely through increased availability of aromatase substrate (Mahendroo *et al.* 1997). However, increased circulating oestradiol is unlikely to explain the adverse metabolic changes observed as reduced, rather than excess, aromatase activity promotes fatty liver and insulin resistance in mouse models, as evident in aromatase-knockout mice (Jones *et al.* 2000).

Ideally, this experiment would have also been performed in gonadectomised male mice to minimise changes in androgens; however, attempts to do this were unsuccessful. Upon gonadectomy, the wild-type mice resisted high-fat diet, neither gaining excess weight nor having raised insulin. Signalling through the androgen receptor (AR) may play a protective role, as disruption of AR predisposes to late-onset obesity (Sato *et al.* 2015), but more so in males than females (when a high-fat diet is required (Fagman *et al.* 2015)). Disruption of hepatic AR also predisposes male but not female mice to hepatic steatosis (Lin *et al.* 2008), emphasising the important role of androgens in metabolic regulation within liver, the major site of 5αR1 expression. Here, female 5αR1-KO mice may be more susceptible to developing steatosis due to higher dependency on small amounts of DHT formed through 5αR1 for AR stimulation than males, who would be protected by their greater pool of testosterone. After inhibition/disruption of 5αR1, excess testosterone may also be converted to oestradiol by aromatase; mice lacking 5αR1 have increased levels of oestrogens (Mahendroo *et al.* 1997), as do men receiving 5αR1 inhibitors (Upreti *et al.* 2014). This is of sufficient magnitude to cause parturition defects in 5αR1-KO mice (Mahendroo *et al.* 1997) and might attenuate the adverse changes in metabolism in male mice.

In a previous attempt to dissect the contribution of androgens to the metabolic phenotype, pharmacological inhibitors of 5αRs were administered to castrated Zucker rats (Livingstone *et al.* 2015). Under these circumstances, insulin resistance was less marked after 5αR inhibition, but hepatic steatosis was still present. This was interpreted as the hepatic lipid phenotype being driven by accumulation of excess glucocorticoids, whereas aspects of peripheral insulin resistance observed were ameliorated by changes in androgen metabolism. The data in female 5αR1-KO mice support the adverse changes in metabolism being driven by glucocorticoids.

In conclusion, studying female mice as a ‘low androgen’ state shows that changes in sex steroid metabolism are not the primary drivers of adverse changes in metabolism observed after deficiency or inhibition of 5αR1 and supports the notion that 5αR1 plays a critical role in regulating tissue glucocorticoid action. Variations in 5αR1 activity in obesity may have therefore have important consequences for onset and progression of metabolic liver disease, and these may be more influential in women or hypogonadal men. Women may be particularly susceptible to adverse metabolic effects of dual 5α-reductase inhibitors, should they be prescribed for hirsutism in the future (Azzouni *et al.* 2013). Close monitoring of insulin sensitivity in these patient subgroups when receiving dual 5αR inhibitors may be necessary.

## Supplementary data

This is linked to the online version of the paper at http://dx.doi.org/10.1530/JOE-16-0125.

## Declaration of interest

The authors declare that there is no conflict of interest that could be perceived as prejudicing the impartiality of the research reported.

## Funding

This research was supported by the Wellcome Trust (072217/Z/03/Z), the British Heart Foundation (FS/08/063 and FS/08/065) and a PhD scholarship from the Carnegie Trust.

## Author contribution statement

The study was designed by D E W L, B R W and R A. The study was performed by D E W L, E M D and T C S M. The manuscript was written by D E W L and R A and revised by B R W, with contributions from all co-authors. R A guarantees the contents.
